# Unequivocal Identification of 1-Phenylethyl Acetate in Clove Buds (*syzygium aromaticum* (L.) Merr. & L.M.Perry) and Clove Essential Oil

**DOI:** 10.3390/foods6070046

**Published:** 2017-06-27

**Authors:** Klaus Gassenmeier, Hugo Schwager, Eric Houben, Robin Clery

**Affiliations:** 1 Givaudan (Schweiz) AG, Dübendorf 8600, Switzerland; Hugo.Schwager@givaudan.com (H.S.); Robin.Clery@givaudan.com (R.C.); 2 Givaudan Nederland BV, GP Naarden 1411, Netherlands; Eric.Houben@givaudan.com

**Keywords:** 1-phenylethyl acetate, styrallyl acetate, alpha methyl benzyl acetate, clove, *Syzygium aromaticum*, volatile composition

## Abstract

The natural occurrence of 1-phenylethyl acetate (styrallyl acetate) was confirmed in commercially available dried clove buds and also in the hydrodistilled oil from clove buds. This confirms previous reports and other anecdotal evidence for its occurrence in nature.

## 1. Introduction

1-Phenylethyl acetate (FEMA: 2684, CAS 93-92-5) is a well-known flavor constituent admitted in many countries as a flavor ingredient. Its flavor has been described as sweet and fruity, tropical, mango, woody, musty, honey like with floral powdery nuances [1]. It is available in artificial and natural versions from various suppliers, and may also be known as styrallyl acetate or alpha methyl benzyl acetate.

According to the Volatile Compounds in Food database [2], its occurrence in food is reported in avocado [3], honey [4], melon [5] and strawberry guava [6]. Other publications state its occurrence in pineapple [7] and banana [8]. Nevertheless, none of the cited publications provides sufficient evidence for the occurrence of the title compound in food, especially when the criteria of the International Organisation of the Flavour Industry—Working Group of Methods and Analysis (IOFI WGMA) recommendation [9] are considered, which require use of an authentic standard and two independent methods of identification, e.g., mass spectrum and retention index. The occurrence of 1-phenylethyl acetate (1-PA) is mentioned in steam-distilled and expressed clove bud oil by Brian Lawrence [10] and in several Cinnamonum species [11], but no analytical details are given in those publications. This has led to remaining doubts about its occurrence in nature and some analytical service labs would regard 1-PA as not yet having been identified in nature. Consequently, flavors containing 1-PA would be regarded as not natural, regardless of whether 1-PA had been obtained from a natural source or not.

Since we had prior evidence in-house of the occurrence of 1-PA in a clove essential oil (un-published results), we re-investigated clove bud concerning the occurrence of the title compound following the recommendation in [9].

## 2. Materials and Methods

Clove buds were obtained from a local retail store. Sample A: Clove buds, whole (Migros, Dübendorf, Switzerland). The exact country of origin could not be given by the supplier, but would be Madagascar, Indonesia, Sri Lanka or Comores (communication Remo Kessler, Customer service, M-Infoline, Zurich, Switzerland). Sample B: Clove buds, whole, organic (Coop, Volketswil, Switzerland), Origin: India and/or Sri Lanka. Sample C: Clove buds, ground (Coop, Volketswil, Switzerland), Origin: Madagascar.

1-Phenylethyl acetate, eugenol, eugenol acetate and beta-caryophyllene were obtained from Aldrich, Switzerland.

Essential oil isolation and analysis: Clove buds (Sample A; 55 g) were finely ground, mixed with water (250 mL), placed in a round bottom flask (500 mL) and hydrodistilled using a Clevenger type apparatus. Distillation was conducted for 1 h and the oil collected in an oil receiver. The essential oil was recovered from the condenser. A dilution 1:5 in methyl tert.butyl ether was injected in the gas chromatograph (GC) with a split ratio of 1:20.

Solid Phase Micro Extraction (SPME): 0.5 g of clove buds were finely ground and sealed in a 20 mL headspace vial. The headspace in the vial was extracted by automated SPME using a CTC autosampler (Agilent, Santa Clara, CA, USA). Equilibration at 40 °C for 10 min, then exposure of the SPME fiber (50/30 µm DCB/CAR/PDMA, Supelco; Bellafonte, PA, USA) for 10 min. The fiber was desorbed directly in a split/splitless inlet (230 °C) for 30 s in splitless mode.

Gas Chromatography Mass Spectrometry: An Agilent Gas Chromatograph 6890 coupled to a mass selective detector (MSD) 5975B and a flame ionization detector (FID) was used. The effluent was split 1:1 between FID and MSD. Linear retention index was calculated on an alkane scale. Column: CP-WAX 60 m × 0.32 mm i.d. × 0.25 µm film thickness (Agilent, Santa Clara, CA, USA). Temperature program: from 35 °C, held for 2 min, to 250 °C at 4 °C/min. Carrier gas: He, linear velocity: 25 cm/s. Injection temperature: 250 °C. Injection volume: 1 µL. Injection mode: split (20:1). FID (250 °C). MS Interface temperature: 280 °C; MS mode: EI at 70 eV; Ion source temperature: 150 °C; Mass range 29–250 u. Data handling was carried out using MSD Chemstation (Agilent, Santa Clara, CA, USA).

Chiral separation was conducted using a β-DEX 120 column (Sigma-Aldrich, Saint Louis, MO, USA, 30 m × 0.25 mm × 0.25 µm film thickness, Nr. 24304a). Carrier gas was helium at 25 cm/s. Temperature program: from 60–130 °C at 2 °C/min then raised to 200 °C at 40 °C/min. The enatiomeric excess was determined by integrating *m/z* 104 and *m/z* 105. For both isomers, clean mass spectra were obtained after background subtraction.

Identification: Mass spectra and retention index were compared with reference samples.

## 3. Results

**Figure 1:**
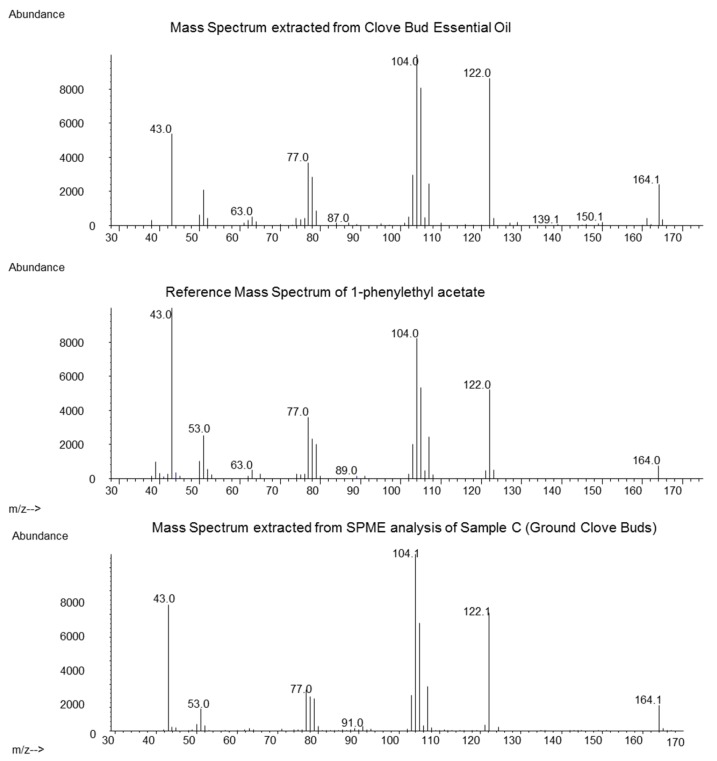
Comparison of the mass spectrum obtained from clove bud essential oil (top), reference mass spectrum from 1-phenylethyl acetate (1-PA) (middle) and solid phase micro extraction (SPME) of ground clove buds (sample C, bottom).

Analysis of the obtained hydrodistilled oil (Sample A) revealed Eugenol (86.3%), beta-Caryophyllene (6.3%) and Eugenyl acetate (4.1%) as the main constituents. For the identification of the 1-PA the relevant retention time window was screened for the occurrence of the most typical mass fragments *m/z* 104, 122 and 164. At a retention time of 31.26 min, the mass spectrum in Figure 1 (top) was obtained after background subtraction. It matched the mass spectrum of the reference (Figure 1, middle). The measured retention index was 1697 compared to 1694 of the authentic reference. Based on the FID signal integration, the area percent of 1-PA was estimated to be around 0.05%. This was a significantly higher value compared to a previous in-house analysis, where ground clove bud was extracted and 1-PA showed a concentration of 0.0027% (internal communication). Lawrence [10] reported the sum amount of 1-PA and two other co-eluting constituents in distilled clove, and in expressed oil from Madagascar at 0.1% and 0.21%, respectively, which closely matches our measured value of 0.05%.

The heating during distillation may introduce chemical changes to a product, and essential oils may contain compounds not present in the native plant. To exclude this possibility, we conducted a SPME extraction of clove bud samples A, B and C. In those three samples, we could also detect the mass spectrum of 1-PA at the correct retention index (mass spectrum of 1-PA from SPME of sample C see Figure 1, bottom). The highest intensity was found in sample C (origin Madagascar), but no quantification was conducted.

The formation pathway of 1-phenylethyl acetate is unclear; there could be an enzymatic or chemical process. The enzymatic processes of biosynthesis often lead to products with an enantiomeric excess of one stereo isomer. To elucidate this, we conducted a chiral analysis of the 1-phenylethyl acetate. Peak identification was based on mass spectrometry and comparison to a sample of 1-PA racemate. In the essential oil obtained from clove buds, we found a significant enantiomeric excess of 62% for the first eluting enantiomer. Lacking enantiomeric pure 1-PA as reference for either of the two 1-PA enantiomers, we could not assign the configuration (R or S) of the major isomer.

## 4. Discussion and Conclusions

1-Phenyethyl acetate was unequivocally identified in clove bud essential oil and in ground clove buds. In lab-distilled essential oil obtained from clove buds, we found an enantiomeric excess of 62% of the first eluting enantiomer. On the basis of these observations, we conclude that 1-phenylethyl acetate occurs naturally in clove buds and is probably the product of enzymatic biosynthesis, and not an artifact of subsequent processing.
